# Integrated endotoxin-adsorption and antibacterial properties of platelet-membrane-coated copper silicate hollow microspheres for wound healing

**DOI:** 10.1186/s12951-021-01130-w

**Published:** 2021-11-22

**Authors:** Zaihui Peng, Xiaochun Zhang, Long Yuan, Ting Li, Yajie Chen, Hao Tian, Dandan Ma, Jun Deng, Xiaowei Qi, Xuntao Yin

**Affiliations:** 1 Department of Breast Surgery, Southwest Hospital, Army Medical University, Chongqing, 400038 China; 2 Department of Radiology, Guangzhou Women and Children’s Medical Center, Guangzhou Medical University, Guangzhou, 510005 China; 3 Institute of Burn Research, Southwest Hospital, State Key Lab of Trauma, Burn and Combined Injury, Chongqing Key Laboratory for Disease Proteomics, Army Medical University, Chongqing, 400038 China

**Keywords:** Bacterial infection, Wound healing, Toxin adsorption, Mesoporous copper silicate microspheres, Photothermal therapy

## Abstract

Serious infection caused by drug-resistant gram-negative bacteria and their secreted toxins (*e.g.*, lipopolysaccharide) is a serious threat to human health. Thus, treatment strategies that efficiently kill bacteria and reducing the impact of their toxins simultaneously are urgently required. Herein, a novel antibacterial platform composed of a mesoporous copper silicate microsphere (CSO) core and a platelet membrane (PM) shell was prepared (CSO@PM). CSO@PM specifically targets bacteria owing to formyl peptide receptors on the PM and, combined with photothermal therapy (PTT), exhibits highly effective bacter icidal activity. Importantly, CSO@PM can adsorb lipopolysaccharide secreted by gram-negative bacteria, resulting in inflammation reduction. Thus, CSO@PM stimulates re-epithelialization and granulation-tissue formation, promoting wound healing. Moreover, this antibacterial platform exhibits no obvious toxicity at all the test concentrations in vitro and in vivo. Thus, CSO@PM exhibits a robust antibacterial effect and a strong toxin-adsorption capacity, facilitating the clinical treatment of many bacterial infections and the development of next-generation antibacterial nanoagents.

**Supplementary Information:**

The online version contains supplementary material available at 10.1186/s12951-021-01130-w.

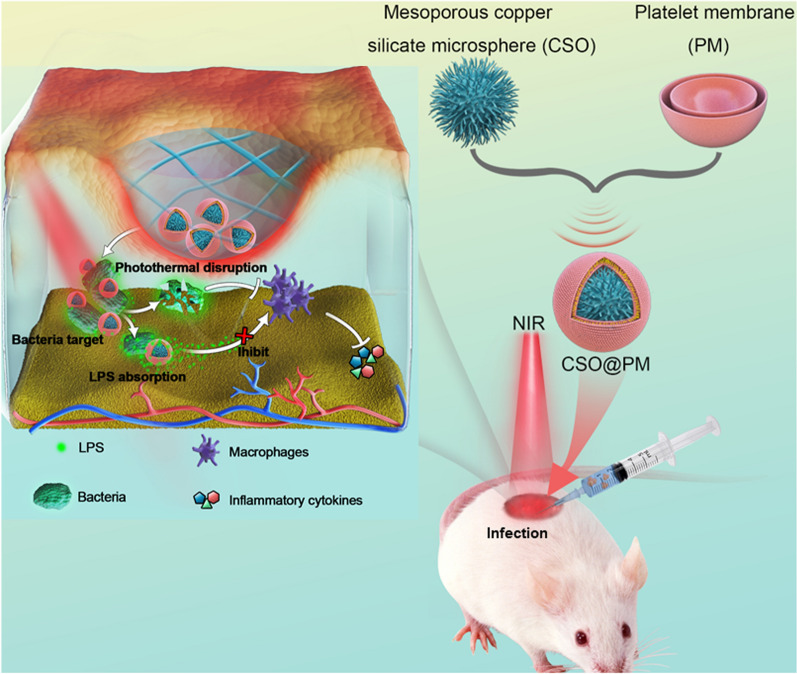

## Introduction

The treatment of bacterial infection is a global challenge that costs millions of dollars per year, presenting a tremendous economic burden for society and patients [1–3]. A broad range of antibiotics are used for treating these infections. However, the widespread use of broad-spectrum antibiotics is making the problem of bacterial resistance an increasingly serious global threat [4, 5]. Compared to Gram-positive bacteria, Gram-negative bacteria show more antibiotic resistance owing to their powerful antimicrobial resistance systems and bilayer membrane structure. In addition, Gram-negative bacteria also secrete toxins such as lipopolysaccharides (LPS), which are highly toxic inflammatory and pyrogenic substance [6]. LPS induce the release of a large number of inflammatory mediators, inducing continuous inflammation at the infected site and thus inhibiting wound healing, leading to chronic wounds [6, 7] and, in extreme cases, life-threatening sepsis [8, 9]. Accordingly, it has been demonstrated that inhibiting bacterial toxin production can reduce the severity of infections [10]. Thus, alternative antibacterial agents that both effectively kill bacteria and remove their secreted toxins are urgently required.

Recent studies have indicated that nanomaterials with photothermal capabilities constitute a new class of therapeutic agents for antibacterial therapy [11–13]. By virtue of their photothermal activity, these materials convert absorbed light energy into heat energy, thereby killing bacteria, minimizing the misuse and abuse of antibiotics. For example, CuS-based photothermal therapy (PTT) is a promising antiseptic strategy to combat bacterial infections [14–17]. In recent years, several research groups, including the authors’, have developed nanomaterials including functionalized graphene and gold nanorods among others, to kill bacteria [18–20]. However, these strategies do not address the toxins secreted by bacteria. Furthermore, specifically targeting bacteria as a means to reduce damage to normal tissues is also a great challenge.

Platelets derived from bone marrow stem cell lineage megakaryocytes, contain numerous growth factors, chemokines, and proteases as well as other proteins that play important roles in hemostatic functions. However, an increasing body of evidence suggests that platelets have several functions beyond hemostasis [21, 22]. For instance, there is abundant evidence for the role of platelets in angiogenesis [23–25] and tissue regeneration [26], while platelet transfusion has been demonstrated to be beneficial for reducing sepsis [27, 28]. Furthermore, the critical roles of platelets in bacterial infection and LPS-induced inflammation have been reported [29, 30]. The multiple functionalities of platelets stem from their unique surface characteristics. Their surfaces are activated to express formyl peptide receptors (FPR), Toll-like receptors (TLR), and chemokine receptors to detect bacterial-related molecular patterns and target bacteria [31, 32], and express Glycoprotein VI (GPVI) and C-type lectin–like receptor 2 (CLEC-2), which are key mediators of inflammation [33]. Given the diversity functions of platelets and the close relationship with inflammation and bacterial infections. And the toxins secreted by bacteria tend to insert into the liposome membranes and form pores, through which liposome adsorb the toxins [34, 35]. The main component of platelet membrane is liposome, which can be used for adsorbing toxins. Thus, wrapping therapeutic nanoparticles (NPs) with PM is a potential strategy to realize simultaneous high-efficiency sterilization and adsorption of bacterial endotoxins.

**Fig. 1:**
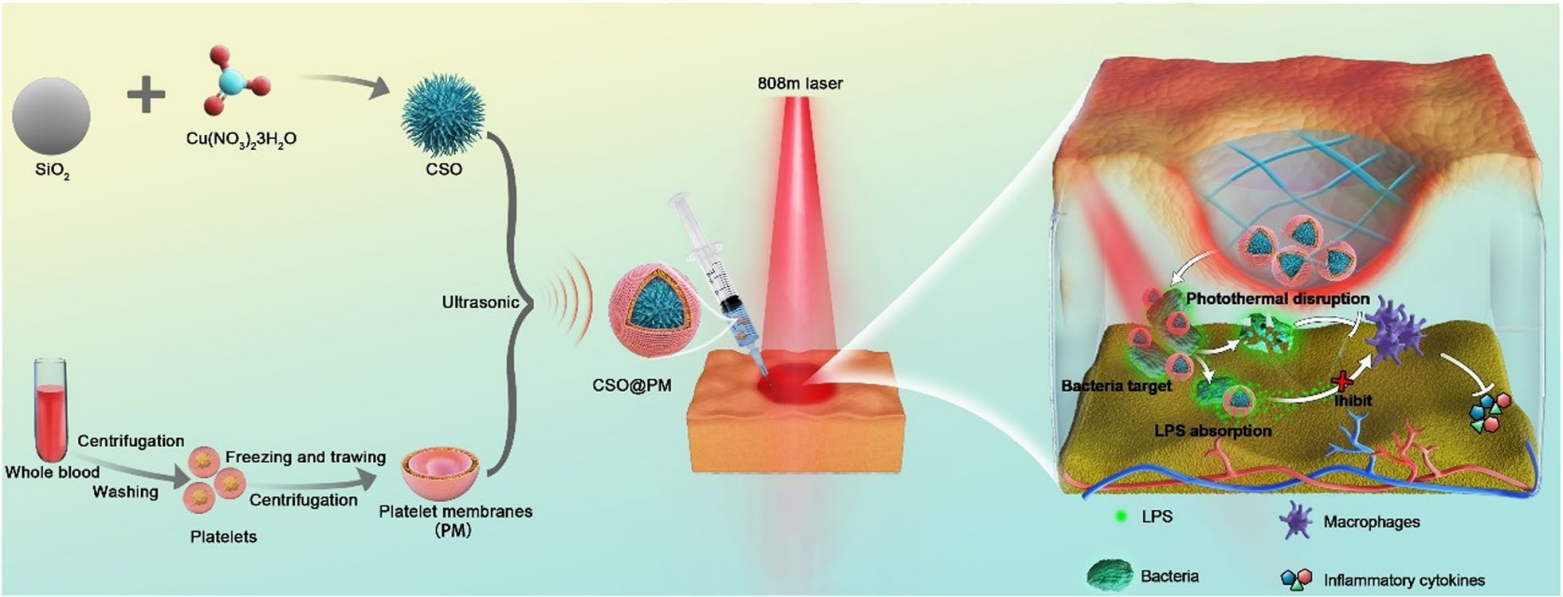
Schematic showing the preparation of CSO@PM NPs and the treatment of wounds infected with multi-drug-resistant Gram-negative bacteria. CSO@PM NPs specifically target bacteria, adsorbing their LPS and, in combination with NIR laser irradiation, effectively kill them, reducing inflammatory reactions and ultimately promoting wound healing

Here, a multifunctional antibacterial agent (CSO@PM) was prepared by coating mesoporous copper silicate microspheres (CSO) with PM assisted by ultrasound irradiation (Fig. 1). CSO@PM exhibits excellent PTT performance [36] and specifically binds to bacteria through formyl peptide receptors, TLRs, and chemokine receptors, enhancing its bactericidal effects [31, 32]. The mesoporous structure of CSO allows the PM to efficiently adsorb the toxins secreted by bacteria, significantly decreasing wound inflammation. Furthermore, copper and silicon ions promote re-epithelialization and collagen deposition during wound healing [37–40]. The Gram-negative, multi-drug-resistant bacteria *Pseudomonas aeruginosa* (*P. aeruginosa*) infected wound model and LPS-induced inflammation wound model were used to study the antibacterial and anti-inflammatory effects of CSO@PM, respectively, demonstrating that that it targets bacteria and, under the action of near-infrared (NIR) light irradiation, exhibits effective bactericidal activity both in vitro and in vivo. Additionally, CSO@PM effectively adsorbs LPS, leading to inflammation reduction in vivo, and stimulates re-epithelialization and granulation-tissue formation, promoting wound healing. Hence, this work provides a feasible strategy for efficient treatment of wounds infected with multi-drug resistant bacteria.

## Materials and methods

### Materials

Tetraethoxysilane (TEOS), ammonium hydroxide (NH_3_·H_2_O, 28%), cupric nitrate trihydrate (Cu(NO_3_)_2_·3H_2_O), and lipopolysaccharide (LPS) were purchased from Sigma-Aldrich (Shanghai, China), Cefoperazone (CFP, CAS: 62893-19-0) was purchased from Macklin. All reagents were applied as received with no further purification.

BALB/c mice (male, 20–25 g) were provided by the Laboratory Animals Department of the Army Medical University (AMU, Chongqing, China). All animal experiments were performed according to ethical standards and with the approval of AMU's Institutional Animal Care and Use Committee. The experimental mice were housed individually in plastic cages with free access to autoclaved standard rodent chow and water under standard conditions (Room temperature: 25 °C; relative humidity: 50%; circadian rhythm: 12 h). All mice were fed adaptively for 1 week before the experiment.

Multi-drug resistant *Pseudomonas aeruginosa* (*P. aeruginosa*, ATCC 27853) was provided by the Clinical Microbiology Laboratory, Institute of Burn Research, Southwest Hospital, AMU (Chongqing, China). NIH 3T3 cells and RAW264.7 cells were provided from the Chinese Academy of Science Cell Bank for Typical Culture Collection.

### Human platelet isolation and membrane derivation

Whole blood samples were collected in tubes containing heparin anticoagulant from human blood with approval from the Ethics Committee of the AMU. Platelets were separated from whole blood by centrifugation and washing. Then, 1 mL aliquots of platelet solution containing ~ 1.5 × 10^9^ platelets were prepared and used to coat the NPs. PM was derived by a repeated freeze–thaw process. Aliquots of platelet suspensions were first frozen at − 80 °C, thawed at room temperature, and pelleted by centrifugation at 4000×*g* for 3 min. The pellets were washed three times with phosphate-buffered saline (PBS) solution containing a protease inhibitor, then subjected to ultrasound treatment (5 min, 42 kHz, 100 W).

### Synthesis of CSO@PM

CSO was synthesized by a facile hydrothermal procedure involving a chemical template-etching process. First, SiO_2_ colloidal spheres were prepared according to the Stöber method [36] and used as a self-sacrificing template. The as-prepared SiO_2_ spheres (0.13 g) were then homogeneously dispersed in distilled water (50 mL) followed by the addition of Cu(NO_3_)_2_·3H_2_O (0.7 mmol, 0.17 g) and NH_3_·H_2_O (AR 25%–28%, 5 mL). After vigorously stirring for 30 min, the resulting suspension was transferred to a Teflon-lined autoclave (50 mL) and maintained at 140 °C for 12 h. The obtained precipitates were collected, washed with distilled water and ethanol, and finally dried at 60 °C. A PM suspension was mixed with an equal volume of CSO solution under ultrasound treatment (5 min, 42 kHz, 100 W). The CSO@PM was finally collected by high-speed centrifugation.

### Characterization of CSO@PM

The morphologies of the CSO@PM NPs were observed by transmission electron microscopy (TEM, JEM-2100F, JEOL, Japan) and scanning electron microscopy (SEM, SU8010, Hitachi, Japan). The phase composition of the NPs was determined by X-ray diffraction analysis (XRD, Rigaku D/Max-2550 V, Geiger flex, Japan). Fourier-transform infrared (FTIR) spectra were recorded on Prestige-21 spectrophotometer (Shimadzu, Japan). The zeta potentials of the samples were measured using a particle characterization system (Zetasizer Nano ZSP, Malvern Instruments Ltd, UK). The optical absorption of CSO was measured using UV–Vis-NIR spectroscopy (UV-3600, SHIMADZU, Japan).

### In vitro biocompatibility of CSO@PM

Cytotoxicity analysis of the material was carried out using 3T3 cells. 3T3 cells were seeded into 96-well culture plates at a density of 1 × 10^4^ cells/well and incubated at 37 °C and 5% CO_2_ for 24 h. Then, the cell culture medium was aspirated and fresh high-glucose Dulbecco's modified eagle medium (DMEM) containing 10% calf serum, 100 U mL^−1^ penicillin, and 100 μg mL^−1^ streptomycin with various concentrations of CSO and CSO@PM (0.025–0.3 mg mL^−1^) were added and incubated at 37 °C for 24 h. Cells were gently washed once with sterile PBS and then treated with 100 μL fresh culture medium and 10 μL cell counting kit-8 (CCK-8, Dojindo, Japan) solution before further incubation at 37 °C for 4 h. The cell viability was then quantified by measuring absorbance at 450 nm using a microplate reader (Thermo Varioskan Flash, USA).

Hemolysis assays were performed on the basis of previously reported methods with some modifications [41]. All the animal experiments were carried out under the approval of the Institutional Animal Care and Use Committee of the Army Medical University. Fresh whole-blood samples were collected from the orbital venous of healthy BALB/c mice. The collected blood samples were centrifuged for 15 min at 231 × *g* to collect erythrocytes and then washed gently three times with saline solution. Then, 3.67 mL saline solution was added to the erythrocytes collected from 1 mL blood, and 100 μL of the diluted erythrocytes was mixed with 1 mL CSO and CSO@PM at various concentrations (100–500 μg mL^−1^). The mixed dispersions were incubated for 3 h at 37 °C and then centrifuged for 15 min at 13,800 × *g* before measuring hemolysis. The hemolysis ratio was quantified by measuring the absorbance value of the supernatant at 540 nm with a microplate reader. Deionized water and saline solution were used as the positive and negative controls, respectively.

$$\mathrm{Hemolysis ratio }(\mathrm{\%})=\frac{{A}_{G}-{A}_{N}}{{A}_{P}-{A}_{N}}\times 100\mathrm{\%}$$

where *A*_*G*_ is the absorbance of the experimental group, *A*_*N*_ is the absorbance of the saline control, and *A*_*P*_ is the absorbance of the water control.

The in vivo biosafety of CSO@PM was evaluated using BALB/c mice (20–25 g, 6–8 weeks). Briefly, 500 μL CSO@PM solution (500 μg mL^–1^) was injected through the tail vein. Mice treated with the same volume of PBS were used as a control group. After being treated with CSO@PM or PBS for 21 days, the mice were sacrificed and their formalin-fixed organs, including the heart, liver, spleen, lung, and kidney, were subjected to histological examination using an optical microscope (VLSM-808-B, Connet, Shanghai Hanyu Optical Fiber Co. Ltd., China). In addition, blood samples were collected and analyzed according to the standard procedures of serum biochemistry [42].

### In vitro targeting properties and antibacterial activity of CSO@PM

Multi-drug-resistant *P. aeruginosa* was employed to investigate the targeting properties and antibacterial effects of CSO@PM. A single bacterial colony on an agar nutrient plate was picked, transferred to 4 mL of Luria–Bertani (LB) medium, and shaken (37 °C, 200 rpm) until the logarithmic growth phase. Then, the cultured bacterial solution was centrifuged (4500 rpm, 6 min), the supernatant was discarded, and the bacteria were washed twice with sterile PBS to remove excess medium. Then, the bacteria were re-suspended in sterile PBS, and the absorbance at 600 nm was adjusted to 0.4–0.5, the corresponding bacterial concentration of which was 10^7^–10^8^ CFU mL^−1^. These bacteria were then used in subsequent in vitro experiments.

To assess the targeting properties of CSO@PM, the test nanomaterials were co-cultured with bacterial suspension in PBS (pH 7.4) for 4 h. The morphologies of the bacteria were revealed by SEM (Inspect F, Royal Dutch Philips Electronics Ltd., Netherlands). The amounts of nanomaterials bound to the bacteria were also determined by inductively coupled plasma–mass spectrometry (ICP–MS):

$${C}_{x}(\mathrm{mg}/\mathrm{kg})=\frac{{C}_{x}(\mathrm{mg}/\mathrm{L})\times \mathrm{f}\times {V}_{0}(mL)\times {10}^{-3}}{\mathrm{m}\mathbf{G}\times {10}^{-3}}$$

$$W(\mathrm{\%})=\frac{{C}_{x}(\mathrm{mg}/\mathrm{kg})\times {V}_{0}(mL)}{{10}^{6}}$$

where *V*_0_ represents the volume of the sample (mL), f represents the dilution multiple of the sample, and *C*_O_ represents the concentration of the element (mg L^–1^), which is obtained experimentally. *C*_*x*_ represents the final result for the measured element (mg kg^−1^) and *W* represents the final result for the measured element.

To investigate the antibacterial activity of CSO@PM, bacterial suspensions were supplemented with different concentrations (10–50 μg mL^–1^) of NPs in 96-well culture plate and illuminated with a laser (808 nm, 1.5 W cm^−2^) for 5 min followed by shaking at 37 °C for 4 h. Then, 20 μL of the bacterial suspension was evenly spread on an LB nutrient plate and incubated at 37 °C overnight. Finally, the plates were photographed and the colony numbers were counted by an automatic colony counter (Supcre, Shineso, China). Bacterial viability was calculated according to the following formula:

$${\text{Bacterial viability }}\left( \% \right) \, = \, \left( {{\text{I}}/{\text{R}}} \right) \, \times { 1}00$$

where I is the experimental group colony count and R is the control colony count.

The adsorption of LPS in in vitro.

### The adsorption of LPS in in vitro

Briefly, 200 μL of 1 ng mL-1 LPS in PBS and different concentrations of NPs were incubated at 37 °C with gentle shaking for 1 h. Then, the mixed solution was centrifuged (4500 rpm, 6 min), and the endotoxin content in the supernatant was measured. The amount of adsorbed endotoxin was calculated from the differential concentration before and after adsorption. The concentrations of endotoxin in samples were measured as follows: samples were diluted to appropriate concentrations of gradients by pyrogen-free water and then the Chromogenic End-point Tachypleus Amebocyte Lysate was added according to the kit introduction. The endotoxin content could be calculated with the standard curve and the absorbance value at 660 nm. All experiments were performed in triplicate.

### The quantification of pro-inflammatory cytokines IL-1β and IL-6

RAW264.7 macrophages were treated with PBS (Control), LPS (100 ng mL^−1^), LPS + CSO (50 μg mL^−1^), and LPS + CSO@PM (50 μg mL^−1^) for 24 h. Total RNA from the treated cells was extracted and transcribed to cDNA, followed by qRT-PCR. The primer sequences for qRT-PCR were shown in Additional file 1: Table S1. After various treatments, the secretion of cytokines IL-1β and IL-6 was quantified by enzyme-linked immunosorbent assay kits (ELISA, Invitrogen) under the guidance of manufacturer’s instructions.

### Therapeutic effect of CSO@PM

Mouse full-thickness skin defect *P. aeruginosa*-infected and LPS-infected wound models were used to evaluate the in vivo antibacterial and endotoxin adsorption effects of CSO@PM and its ability to promote wound healing. First, BALB/c mice were fully anesthetized (1% pentobarbital, intraperitoneal injection) and the dorsal hair of the mice was removed. Then, the back skin of the mouse was disinfected with 75% alcohol and 5-mm full-thickness skin defect wounds were created on the left and right sides using a puncher. Before photographing the wounds, a 5-mm sterilized disc was placed beside the wound to indicate the size of the initial wound and allow comparison.

#### *P. aeruginosa*-infected wounds

A *P. aeruginosa* suspension (20 μL, 2.0 × 10^7^ CFU mL^–1^) was used to infect the tissue of the mice and establish an experimental model of infection. Twenty-four hours later, thirty mice with full-thickness skin defect wounds were randomly divided into six groups (five mice in each group), i.e., CFP group (128 μg mL^−1^), CSO group (50 μg mL^−1^), CSO with NIR irradiation group (CSO + NIR,50 μg mL^−1^, 808 nm, 1.5 W cm^−2^, 10 min), CSO@PM group (50 μg mL^−1^), and CSO@PM with NIR irradiation group (CSO@PM + NIR,50 μg mL^−1^, 808 nm, 1.5 W cm^–2^, 10 min). The temperatures of the wounds were monitored using thermographic images captured with an infrared thermal imaging system (VLSM-808-B, Connet, Shanghai Hanyu Optical Fiber Co., Ltd., China). On the 1th, 3th, 5th, 7th, and 9th day post-surgery, the wounds were administrated and photographed. The area of the wound was calculated using Image J software. The wound tissue removed after the end of treatment on day 7 was used for tissue bacterial analysis. The specific procedure is to break the wound tissue with a homogenizer, and then a standard plate counting method was used to determine the bacterial survival rate (the homogenized tissue was inoculated onto an agar plate and cultured at 37 °C for 24 h before counting).

#### LPS-infected wounds

LPS (20 μL, 200 ng mL^−1^) was added as a droplet to the wound site. Twenty mice with full-thickness skin defect wounds were randomly divided into four groups (five mice in each group), i.e., PBS group (control), CSO group (50 μg mL^−1^), PM group, and CSO@PM group (50 μg mL^−1^). On the 1th, 3th, 5th, 7th, and 9th day post-surgery, the wounds were photographed. The area of the wound was calculated using Image J software. The wound healing rate was calculated according to:

$${\text{Wound healing rate }}\left( \% \right)\, = \,\left( {I{-}R} \right)/I\, \times \,{\text{1}}00.$$

where *I* represent the initial area of the wound and *R* represents the wound area measured after infection.

Real-time fluorescent quantitative PCR was used to analyze the mRNA expression of pro-inflammatory mediators (IL-1β and IL-6). And the levels of pro-inflammatory cytokines (IL-6 and IL-1β) were assessed using ELISA kits according to the manufacturers’ instructions. Optical density was measured at 450 nm and the amount of cytokine or chemokine was calculated from a standard curve prepared with the recombinant protein. The experiments were repeated at least three times independently.

#### Histological analysis of wound tissue

The mice were sacrificed on the day 7 post-surgery, the wound tissues were collected, and tissue sections were prepared by paraffin embedding and subjected to hematoxylin and eosin (H&E) and Masson's trichrome (MT) staining.

#### Statistical analysis

The experimental data are expressed as mean ± standard deviation (SD), and the significant difference between groups was determined using unpaired *t*-test (for two groups) and one-way analysis of variance (ANOVA) (for more than two groups) in Origin 8.5. The statistical significance was set as P < 0.01 (*) and P < 0.001 (**).

## Results and discussion

### Characterizations of the CSO@PM

**Fig. 2:**
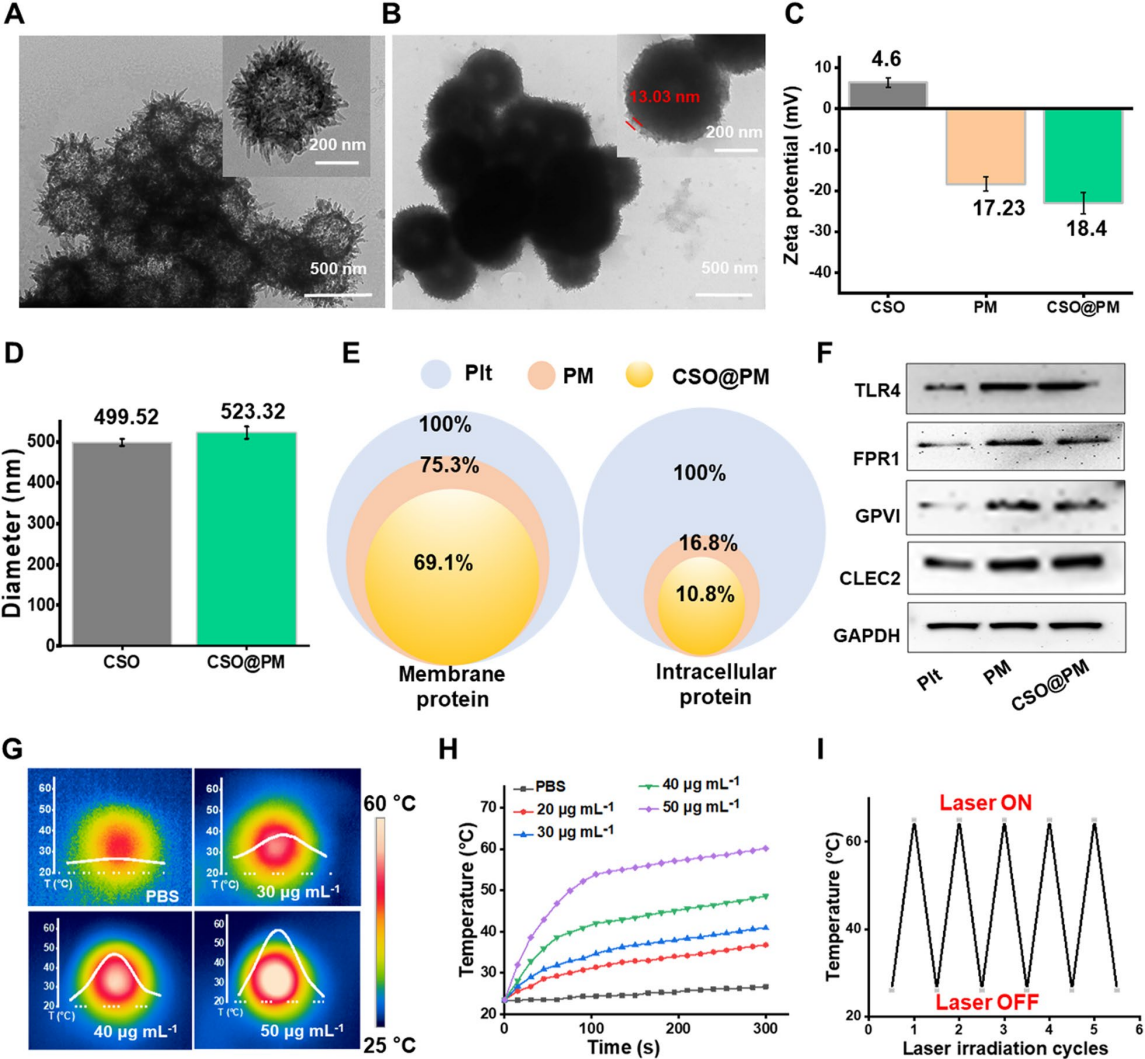
Characterization of CSO@PM. Representative TEM images showing the morphologies of CSO **A** before and **B** after PM coating. **C** Zeta potential values for CSO, PM, and CSO@PM. Data are mean ± SD (*n* = 3). **D** Hydrodynamic diameters of CSO and CSO@PM. Data are mean ± SD (*n* = 3). **E** Preservation of the PM proteins and **E** intracellular proteins detected in platelets, PM, and CSO@PM using proteomic analysis. **F** Western blot analysis of TLR4, FRP1, GPVI, GLEC-2, and GAPDH in platelets, PM, and CSO@PM. **G** Corresponding thermographic images and **H** temperature evolution curves of CSO suspensions with different concentrations in PBS (pH 7.4) under 808 nm NIR laser irradiation at 1.5 W cm^−2^. **I** Thermal response of CSO under repeated laser irradiation (*n* = 5; 808 nm, 1.5 W cm^–2^)

To prepare the CSO@PM, CSO NPs were successfully synthesized by a hydrothermal method using SiO_2_ spheres as a sacrificial template [43] and then underwent encapsulation with PM by an ultrasound-assisted method. As shown in Fig. 2A, the CSO is synthesized as chrysanthemum-like particles with some agglomeration, which is consistent with a previous report [44]. This may be due to the change in the surface charge of silica caused by its hydrolysis under alkaline conditions [45]. Compared with bare CSO, CSO@PM has a clear core–shell structure (Fig. 2A, B, and Additional file 1: Figure S3A, B), where the thickness of the outer shell is ~ 13 nm, indicating that the PM provides sufficient coverage for the NPs (Fig. 2B). The average zeta potential of CSO@PM is − 18.4 ± 1.7 mV, similar to that of PM (− 17.23 ± 2.5 mV) but significantly lower than that of bare CSO (6.4 ± 1.1 mV) (Fig. 2C), indicating the successful coating of the NPs with PM. The average hydrodynamic diameter of CSO@PM is 523.2 ± 14.9 nm (Fig. 2D), which is slightly higher than that of bare CSO (499.5 ± 8.9 nm). These results are consistent with a previous report [46].

The surface areas and pore volumes of CSO@PM were measured by N_2_ physical adsorption–desorption isotherm analysis. As shown in Additional file 1: Figure S1A, the isotherms for CSO@PM present typical type-IV patterns, indicating the presence of mesopores in the materials. The Brunauer–Emmett–Teller surface area of CSO@PM is 138.5 m^2^ g^−1^. The pore size distribution curve of CSO@PM derived from Barrett–Joyner–Halenda analysis revealed an average mesopore size of 4.01 nm (Additional file 1: Figure S1B). The XRD pattern for CSO could be indexed to chrysocolla (Cu_2-X_Si_2_O_5_(OH)_3_·XH_2_O, Additional file 1: Figure S2) without any impurities. It is worth noting that the CSO has a bimodal pore-size distribution, which is due to the effects of its unique structure and hydrothermal treatment. A large pore size is suitable for capturing large biomolecules [47].

To verify whether the synthetic process influences the integrity of membrane proteins, we carried out a proteomic analysis to categorize the quantities of proteins belonging to different cellular components based on Gene Ontology Annotation. Up to 69.1% of the membrane proteins were preserved, while 89.2% of the intracellular proteins were removed during the preparation of CSO@PM (Fig. 2E). To further confirm the retention of functional membrane proteins on the CSO@PM, Western blot analysis was performed. CSO@PM bears specific proteins of the key targeted bacteria (FPR1 and TLR4) and the inflammation mediators GPVI and CLEC-2 (Fig. 2F). The stability of CSO@PM was examined by observing the zeta potential changes over time, and CSO@PM showed negligible zeta potential change for 7 days (Additional file 1: Figure S4). Furthermore, the NPs remain stable in PBS over 7 days at 4 °C (Additional file 1: Figure S5A), and the change in average hydrodynamic diameter for 7 days is also negligable (Additional file 1: Figure S5B). And the western blot analysis also revealed that after storage in PBS, the changes in the specific proteins of the key targeted bacteria (FPR1 and TLR4) and the inflammation mediators GPVI and CLEC-2 for 7 days are negligible (P > 0.05) (Additional file 1: Figure S6). Collectively, these results demonstrate the excellent stability of CSO@PM in biological environments.

Previous studies have demonstrated that CSO strongly adsorbs NIR light [48, 49]. In our study, the UV–Vis-NIR spectra of CSO@PM in PBS showed that the CSO@PM exhibits strong absorbance at ∼808 nm (Additional file 1: Figure S7), making it suitable for photothermal therapy with 808 nm laser irradiation. Clearly, the temperature change of the aqueous CSO@PM solution exhibits a concentration-dependent relationship, and the temperature of the aqueous CSO@PM (50 μg mL^−1^) solution exceeds 65 °C within 10 min at 1.5 W cm^−2^, while the temperature of water without CSO@PM shows no increase (Fig. 2G, H). Furthermore, no noticeable changes in the temperature of CSO@PM are observed after undergoing six cycles of laser irradiation (1.5 W cm^−2^, 10 min, Fig. 2I), indicating its excellent photothermal stability. These results indicate that CSO@PM is a promising candidate for photothermal sterilization.

### In vitro targeting properties and antibacterial activity of CSO@PM

**Fig. 3:**
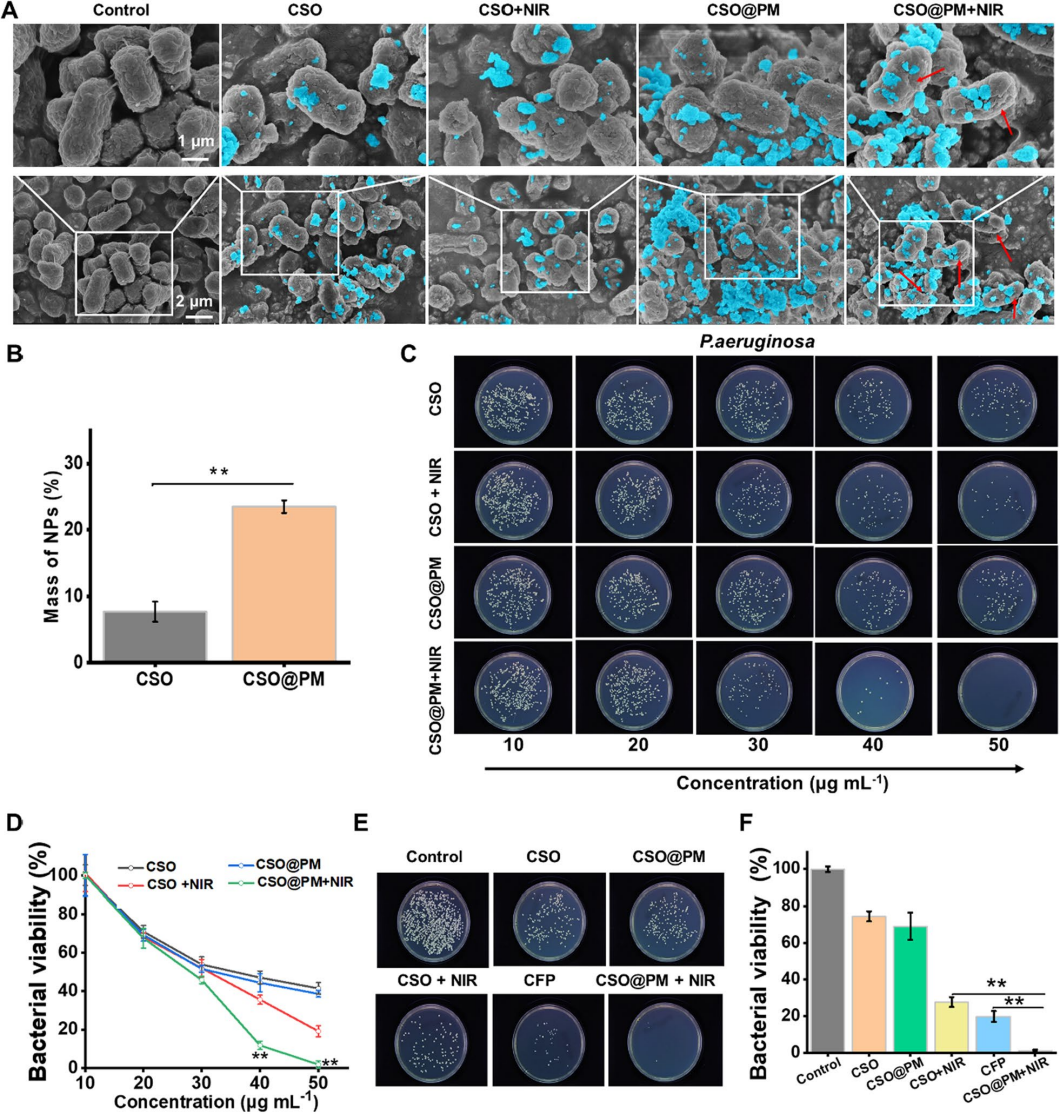
In vitro bactericidal activity of CSO@PM. **A** SEM images for the control (PBS treated), CSO, CSO@PM, CSO + NIR, CSO@PM + NIR groups after incubation with *P. aeruginosa*. Blue indicates NPs. **B** Mass of NPs bound to *P. aeruginosa* after co-incubation with CSO or CSO@PM. Values are mean ± SD (*n* = 3) and ** indicates P < 0.001. **C** Representative images and **D** quantitative analysis of bacterial colonies formed by *P. aeruginosa* after exposure to CSO and CSO@PM with or without 808 nm NIR irradiation. The values are shown as mean ± SD (*n* = 3) and ** indicates P < 0.001 compared with the corresponding control group. **E** Representative images and **F** quantitative analysis of bacterial colonies formed by *P. aeruginosa* after exposure to CFP, CSO, or CSO@PM with or without 808 nm NIR irradiation. The values are shown as mean ± SD (*n* = 3) and ** indicates P < 0.001 compared with the control group

SEM was used to investigate the targeting properties of CSO@PM.As demonstrated in Fig. 3A, we found almost very little CSO NPs on the bacteria, while there was a large amount of CSO@PM NPs bound to the bacteria, suggesting that PM was an effective targeting to bacteria. The EDS element mapping technique analysis provides more supportive evidence for bacterium-targeting properties of CSO@PM (Additional file 1: Figure S8). This may be attributed to PM expressing formyl peptide receptors, TLRs, and chemokine receptors to detect bacteria-related molecular patterns and target bacteria [31, 32]. In order to further investigate the targeting properties of CSO@PM, we incubated CSO and CSO@PM with *P. aeruginosa*. Quantitative ICP-MS analysis showed that 23.5% of the CSO@PM adheres to the *P. aeruginosa* bacteria cells, while the corresponding value for CSO is only 7.69% (Fig. 3B). This demonstrates that CSO@PM has significant bacteria-targeting ability. Additionally, we also observed the morphological changes of *P. aeruginosa* upon co-incubation with CSO@PM. The untreated bacteria remain fully active with typical club-like shapes and intact surfaces. However, the *P. aeruginosa* treated with CSO@PM under 808 nm NIR laser irradiation exhibits a certain degree of distortion and pleats and the cellular walls and membranes has a slight lesion and aperture (Fig. 3A, red arrows). The above results indicate that the PM imparts significant bacteria-targeting properties to CSO NPs. Furthermore, owing to the excellent photothermal properties of copper, CSO@PM can induce bacterial lysis under 808 nm NIR laser irradiation.

To further investigate the antibacterial activity of CSO@PM, in vitro antibacterial assays were performed. *P. aeruginosa* bacteria was chosen as a representative multi-drug-resistant gram-negative because it is resistant to many antibiotics, including carbapenems, which are the most commonly used antibiotics for multi-drug-resistant bacteria [50]. As expected, both CSO (50 μg mL^−1^) and CSO@PM (50 μg mL^−1^) exhibit very weak antibacterial activity against *P. aeruginosa* (Fig. 3C, D). However, when CSO and CSO@PM are exposed to 808 nm NIR laser irradiation, they exhibit strongly enhanced antibacterial effects (Fig. 3C, D). Furthermore, the CSO@PM + NIR treatment has a much higher antibacterial effect than CSO + NIR treatment (P < 0.001), where CSO@PM almost completely kills the bacteria at 50 μg mL^−1^ and CSO only kills ~ 80.8% of the bacteria (Fig. 3C, D). This may be attributed to the bacterial targeting imparted by the PM, making the bactericidal efficiency of CSO@PM + NIR is much higher than that of CSO + NIR. Additionally, we also study the antibacterial effects of our antibacterial agent to multidrug-resistant gram-negative *Klebsiella pneumoniae* (*K. pneumoniae*)and gram-positive *Staphylococcus aureus* (*MRSA*). As shown in Additional file 1: Figure S9, at a concentration of 50 μg mL^−1^, CSO@PM NPs exhibit considerable antibacterial effects when exposed to 808 nm NIR laser irradiation; it almost completely kills the bacteria. Overall, our experimental results clearly indicate that CSO@PM has excellent bactericidal efficiency, which is beneficial for the treatment of drug-resistant infections.

We also compared the antibacterial effects of CSO@PM and cephalosporins, which has been proven to have activity against an expanded spectrum of Gram-negative bacterial infections [51–53]. CFP is one of the most active of these cephalosporins and has a high activity against *P. aeruginosa*. Studies have demonstrated that ~ 80% of *P. aeruginosa* strains are killed by 128 μg mL^−1^ CFP [54, 55]. As shown in Fig. 3E, F, the bactericidal activity of CSO@PM plus laser irradiation against Gram-negative *P. aeruginosa* is significantly higher than that of CFP, and no bacterial regrowth is observed within 24 h (P < 0.001). The results clearly demonstrate that CSO@PM combined with NIR irradiation only requires a drug dose of 50 μg mL^−1^, which is significantly lower than the effective antibacterial concentration of CFP (128 μg mL^−1^) [55]. Overall, these results clearly indicate that CSO@PM has excellent bactericidal efficiency, which is beneficial for the treatment of drug-resistant infections.

### In vitro anti-inflammatory activity of CSO@PM

In view of the benefits of platelet infusion treatment for LPS-induced sepsis [27.28] and the advantages of mesoporous materials with large specific surface areas and pore structure as adsorbents [47], we sought to determine whether CSO@PM can be used as a potential adsorption carrier for LPS.

**Fig. 4:**
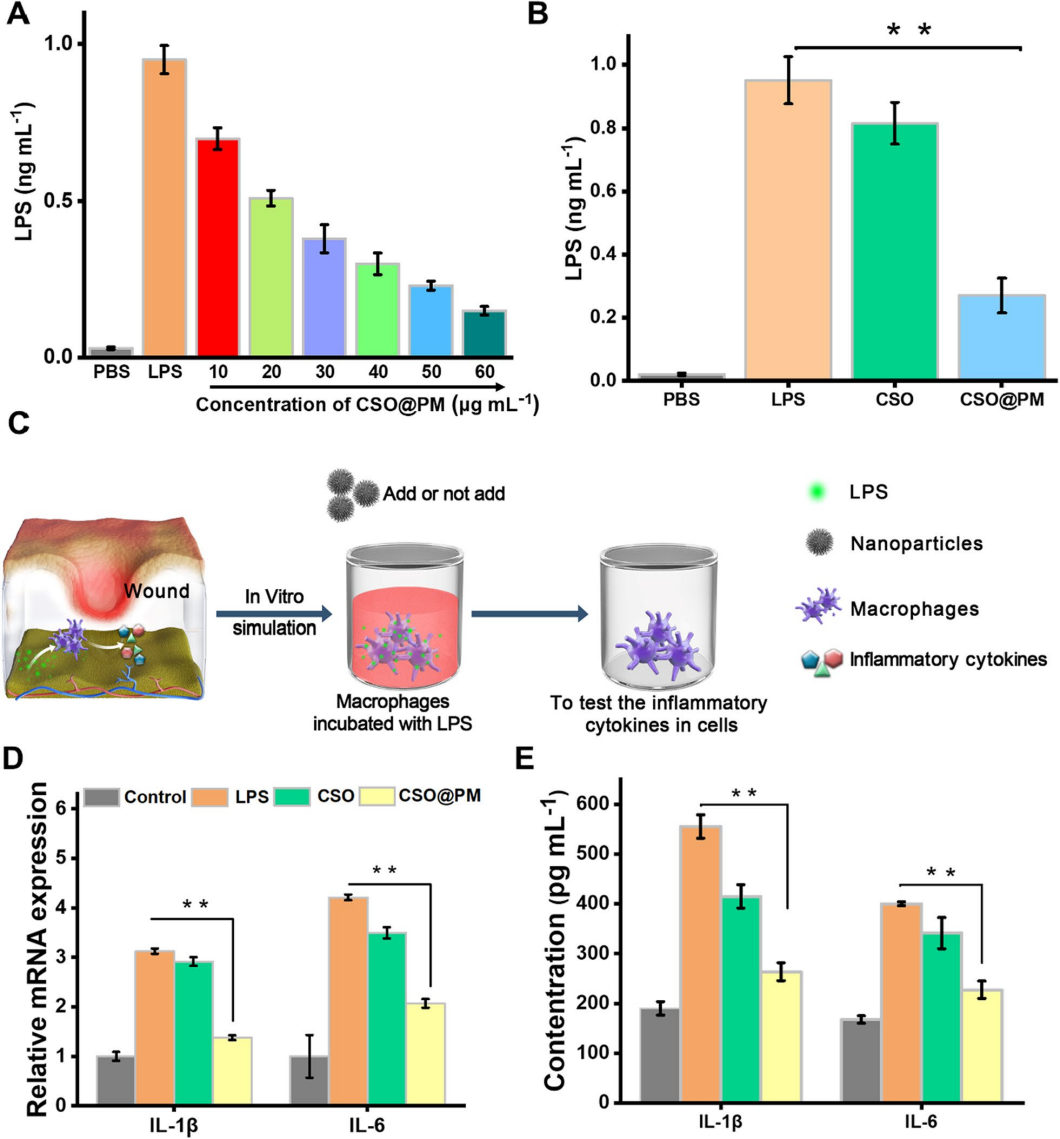
Anti-inflammatory activity of CSO@PM in vitro. **A** LPS adsorption capacity of CSO@PM. The initial concentration of LPS was 1 ng mL^−1^. **B** LPS adsorption for the PBS, LPS (100 ng mL^−1^), CSO (50 μg mL^−1^), and CSO@PM (50 μg mL^−1^) groups. **C** Schematic of the co-culture system composed of RAW246.7 cells, LPS, and CSO@PM. **D** mRNA and **E** protein expression of IL-1β and IL-6 in RAW264.7 macrophages. The values are shown as mean ± SD (*n* = 3) and ** indicates P < 0.001 compared with the corresponding control group

At an LPS concentration of 1 ng mL^−1^, adsorption is positively correlated to CSO@PM concentration (Fig. 4A). Furthermore, the adsorption capacity for the CSO@PM group is significantly higher than that of bare CSO (Fig. 4B). The Additional file 1: Figure S10 provides more supportive evidence for the endotoxin absorption ability of CSO@PM NPs. These results clearly demonstrate that CSO@PM can adsorb LPS, mainly due to the PM on its surface. This suggests that CSO@PM could adsorb bacteria-secreted LPS, thereby alleviating a series of inflammatory responses caused by LPS, and that CSO@PM shows promise as an anti-inflammatory material.

To further investigate the anti-inflammatory activity of CSO@PM, LPS-stimulated murine macrophages (RAW264.7) were used to mimic the inflammatory environment (Fig. 4C) [56]. As shown in Fig. 4D, LPS (100 ng mL^−1^) induces inflammation in RAW264.7 cells, as evidenced by the upregulated mRNA expression of pro-inflammatory cytokines IL-1β and IL-6 in RAW264.7 cells upon LPS stimulation. Compared with the control group (treated with PBS), the mRNA expressions of IL-1β and IL-6 increase 5.07- and 5.22-fold upon LPS stimulation, respectively (P < 0.001). In contrast, treatment with CSO@PM (50 μg mL^−1^) significantly inhibits the LPS-induced expression of IL-1β and IL-6 (P < 0.001).

Next, the effects of CSO@PM on the production of cytokines in LPS-stimulated RAW264.7 cells were investigated using ELISA. As shown in Fig. 4E, for the positive control treated with LPS (100 ng mL^−1^), the concentrations of secreted IL-1β and IL-6 are 555.26 ± 23.46 and 400.18 ± 3.85 pg mL^−1^, respectively, while the addition of CSO@PM (50 μg mL^−1^) to the stimulated cultures suppresses IL-1β and IL-6 secretion to 263.65 ± 17.97 and 227.65 ± 17.40 pg mL^−1^, respectively, which are significantly lower than the corresponding values for the LPS group (P < 0.001).

Secretion of both IL-1β and IL-6 in macrophages is used extensively as a biomarker of inflammation. They are pro-inflammatory cytokines with many functions, including those involved in chronic inflammatory reaction [57]. Thus, our results indicate that CSO@PM exerts anti-inflammatory activity by inhibiting the LPS-induced expression of pro-inflammatory cytokines IL-1β and IL-6 in RAW264.7.

### Therapeutic effect of CSO@PM on *P. aeruginosa*-infected wounds

For in vivo studies, a multi-drug-resistant *P. aeruginosa-*infected murine skin wound model was utilized. *P. aeruginosa* is resistant to a great deal of antibiotics, including carbapenems [50]. Immediately upon the application of CSO@PM and NIR irradiation (1.5 W cm^–2^, 10 min), the temperature of the wound rapidly increases to 56 °C, while the temperature for the control group increases to only 38 °C (Additional file 1: Figure S11A,B). Thus, these data suggest that CSO@PM exhibit remarkable photothermal effects in vivo.

**Fig. 5:**
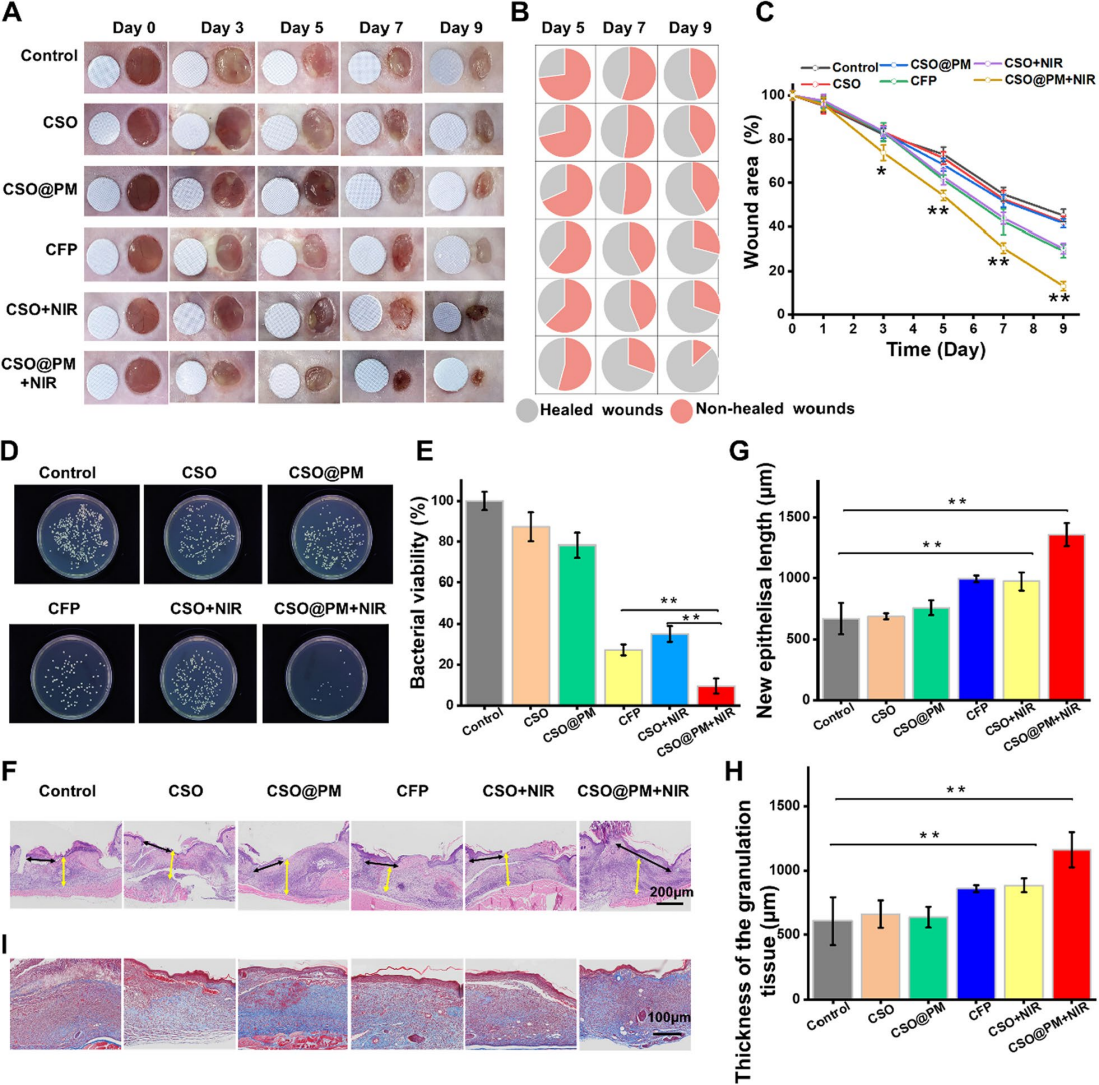
Effects of CSO@PM on the healing of *P. aeruginosa*-infected wounds. **A** Photographs of *P. aeruginosa*-infected wounds under different treatments. The round blue card with a 5 mm diameter indicates initial wound size. **B** Fractions of the wounds healed by the different treatments on days 5, 7, and 9. The values are shown as mean ± SD (*n* = 3). **C** Quantitative analysis of wound area for each group. The values are shown as mean ± SD (*n* = 3). * P < 0.1 and ** P < 0.001 compared to the control group. **D** Photographs and **E** quantitative counts of bacterial colonies formed by *P. aeruginosa* obtained from wound tissues. The values were shown as mean ± SD (*n* = 3). * P < 0.1 and ** P < 0.001 compared to the corresponding control group. **F** H&E staining images of mouse wound tissue from all groups at days 7. Black arrows indicate the length of newly regenerated epidermis. Yellow arrows indicate thickness of granulation tissue. Scale bar: 200 μm. **G** Neo-epidermis length and **H** thickness of granulation tissue data. The values are show as mean ± SD (*n* = 3). * P < 0.1 and ** P < 0.001 compared to the corresponding control group. **I** Masson images of mouse wound tissue from all groups on day 7. Scale bar: 100 μm

To further investigate the effects of CSO@PM in vivo. Infected wounds were treated with PBS (control), CFP (128 μg mL^−1^, the effective inhibitory concentration), CSO (50 μg mL^−1^), CSO@PM (50 μg mL^−1^), CSO (50 μg mL^−1^), or CSO@PM (50 μg mL^−1^), sometimes combined with NIR irradiation (CSO + NIR, CSO@PM + NIR). The NIR irradiation (808 nm) was applied at an intensity of 1.5 W cm^–2^ for 10 min. As shown in Fig. 5A–C, the appearance of wounds and quantitative closed wound areas in the control, CFP, CSO, CSO@PM, CSO + NIR and CSO@PM + NIR groups show that, in the 2 days after treatment, no obvious difference is observed in the closed wound area between all the groups (P > 0.05). However, continuous observation of the wounds revealed that the wound healing for the CSO@PM + NIR group is always significantly better than that of the control group on days 5, 7, and 9 post-surgery (P < 0.001) (Fig. 5A–C). On day 9, the CSO@PM + NIR mice exhibit a healing rate of ~ 90% (Fig. 5C).

To evaluate the actual bactericidal effect of CSO@PM in vivo, the wound tissues were harvested and homogenized to quantify the amount of residual bacteria. As shown in Fig. 5D, E, compared with the CFP and CSO + NIR groups, significantly fewer bacterial colonies are observed in the CSO@PM + NIR group (P < 0.001). These data confirm that CSO@PM combined with NIR irradiation has outstanding antibacterial activity, leading to rapid wound healing. The recovery of re-epithelialization and granulation tissue formation are key factors for evaluating wound healing [58]. Therefore, the wound tissue was collected and H&E stained to investigate the effect of CSO@PM on re-epithelialization and granulation tissue formation (Fig. 5F). As shown in Fig. 5G, H, the neo-epidermis lengths of wounds in the CSO@PM + NIR group were longer than corresponding control group, and the thickness of granulation tissue for the CSO@PM + NIR group are significantly thicker than those for the corresponding control group (P < 0.001). In addition, compared with the corresponding control group, extensive collagen deposition is observed for the CSO@PM + NIR group (Fig. 5I). Previous studies have shown that bacterial infection will extend the inflammatory phase and inhibit collagen deposition at the wound site[59]. Collagen plays an important role in extracellular matrix reorganization and tissue remodeling [60]. The increased collagen expression for the CSO@PM + NIR group facilitated extracellular matrix reorganization, which in turn induced improved re-epithelialization and granulation tissue. However, although bacterial infection and their secreted toxins had a side effect on the wound healing, the CSO@PM + NIR significantly promoted infected skin wound healing in vivo by facilitating both re-epithelialization and granulation-tissue, which were mainly attributed to the efficient bactericidal function of the CSO@PM NPs under 808 nm laser irradiation.

These data show that CSO@PM in combination with NIR irradiation has significant bactericidal activity and can promote wound healing by accelerating the regenerative epithelialization, granulation thickening, and collagen deposition of infected wounds.

### Therapeutic effect of CSO@PM on LPS-infected wounds

**Fig. 6:**
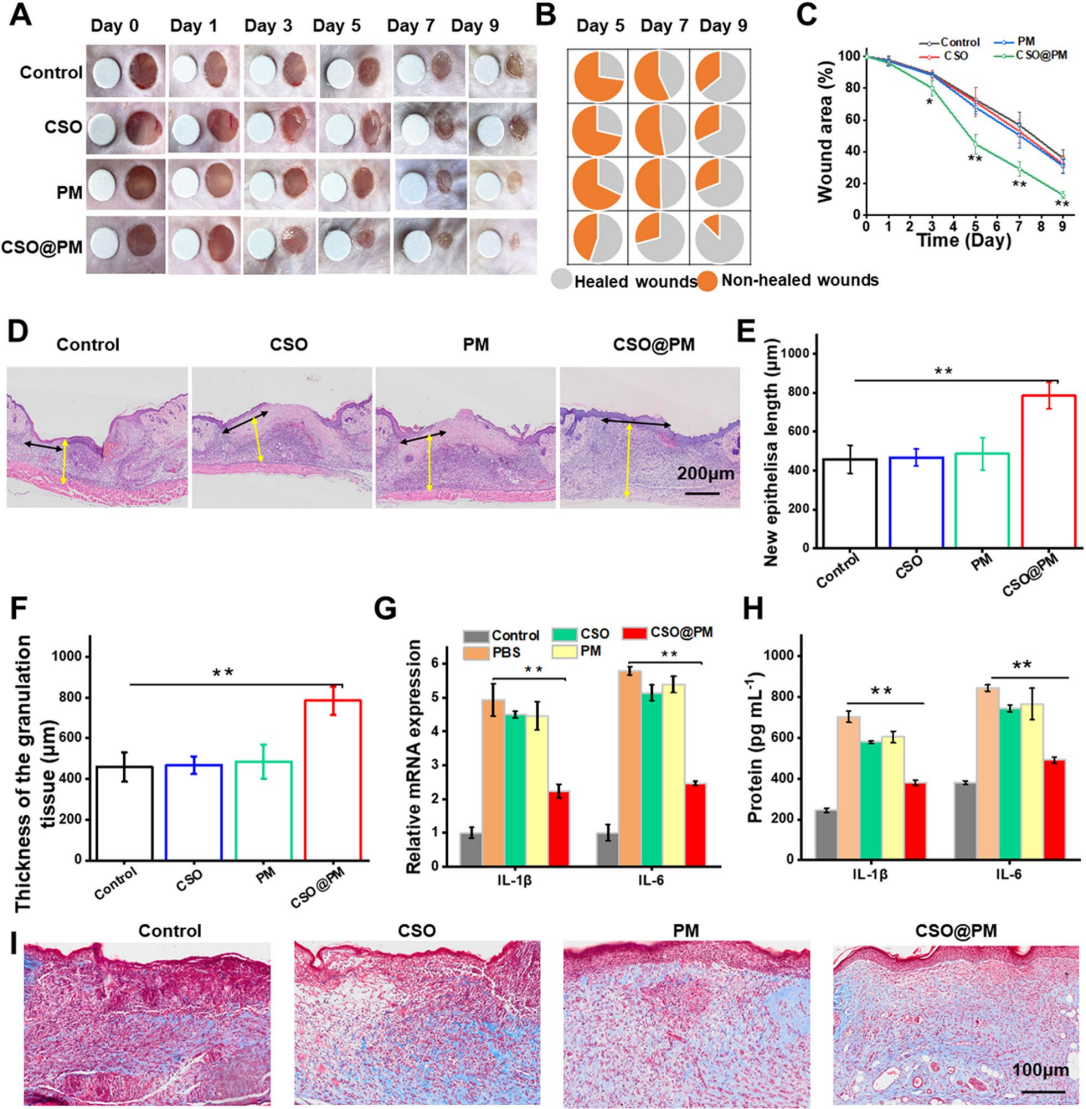
Effect of CSO@PM on LPS-infected wound healing. **A** Photographs of LPS-infected wounds under different treatments. The round blue card with a 5 mm diameter indicates initial wound size. **B** Fractions of the wounds healed by the different treatments on days 5, 7, and 9 (*n* = 3). **C** Quantitative wound area analysis for each group (*n* = 3). * P < 0.1 and ** P < 0.001 compared to the corresponding control group. **D** H&E images of mouse wound tissue from all groups at day 7. Black arrows indicate the length of newly regenerated epidermis. Yellow arrows indicate thickness of granulation tissue. Scale bar: 200 μm **E** Neo-epidermis length and **F** granulation tissue thickness data. **G** Real-time quantitative PCR detection results for IL-1β and IL-6 mRNA and **H** protein expression of IL-1β and IL-6 in LPS-infected wounds at day 7. The values are shown as mean ± SD (*n* = 3). * P < 0.1 and ** P < 0.001 compared to the corresponding control group. **I** Masson images of mouse wound tissue from all groups on day 7. Scale bar: 100 μm

To verify the effect of CSO@PM on LPS-infected wounds, wound healing experiments were conducted in LPS-treated full-thickness skin wound model. The wounds were treated with PBS, CSO (50 μg mL^−1^), PM, and CSO@PM (50 μg mL^−1^). As shown in Fig. 6A, macroscopic analysis of the wound closures showed that, compared with the control group, wounds treated with CSO@PM show significantly faster closure. Continuous observation of wounds showed that on day 9, the CSO@PM-treated wound has undergone ~ 90% wound closure, while the corresponding control groups have closure rates of ~ 54%–65% of the (Fig. 6A–C). The results clearly demonstrate that the healing rate for the CSO@PM group is significantly higher than that of the control (P < 0.001). Although previous studies have revealed that PM may adsorb toxins [61], the healing rate for the LPS-infected wounds treated with PM is significantly lower than that of the CSO@PM group (P < 0.001), as shown in Fig. 6C. This may be because CSO@PM not only adsorbs toxins, it also locks them away in its porous structure. This hypothesis is supported by previously reported results [61].

Again, the wound tissue was collected and H&E staining was performed to investigate the effect of CSO@PM on re-epithelialization and granulation tissue formation (Fig. 6D). As shown in Fig. 6E, F, compared with the control, the granulation tissues for the CSO@PM + NIR group are the thickest and the neo-epidermis lengths are the longest (P < 0.001). In addition, extensive collagen deposition is observed in the wounds treated with CSO@PM (Fig. 6I). These results indicate that CSO@PM has immense promise for treating LPS-infected wounds.

In order to confirm that CSO@PM adsorbs LPS to reduce the inflammatory response in LPS-infected wound to promote wound healing. RNA was extracted from the wound tissues of the control (not infected with LPS) and LPS-infected wounds treated with PBS, CSO (50 μg mL^−1^), PM, and CSO@PM (50 μg mL^−1^). Then, real-time fluorescent quantitative PCR was used to analyze the mRNA expression of pro-inflammatory mediators (IL-1β and IL-6). As shown in Fig. 6G, compared with the control group (treated with PBS), the expression of IL-1β and IL-6 mRNA for the LPS group is significantly increased (P < 0.001). However, CSO@PM treatment significantly inhibits the LPS-induced increase of IL-1β and IL-6 mRNA expression (Fig. 6G). In addition, we also analyzed the expression of cytokines in LPS-infected wounds by ELISA. As shown in Fig. 6H, the IL-1β and IL-6 levels for the LPS group are 704.21 ± 27.84 and 844.59 ± 15.68 pg mL^−1^, respectively. However, the IL-1β and IL-6 levels are decreased to 380.13 ± 11.58 pg and 492.58 ± 14.16 pg mL^−1^, respectively, by CSO@PM treatment. Thus, CSO@PM significantly inhibits the expression of inflammatory biological markers IL-1β and IL-6 [57], which may be a synergistic result of the anti-inflammatory properties of PM [27] and the porous structure of CSO [47].

In general, these data confirmed that CSO@PM inhibits the expression of IL-1β and IL-6 through LPS adsorption, thereby reducing the inflammatory response of the wound and ultimately promoting wound healing.

### In vitro and in vivo biocompatibility

**Fig. 7:**
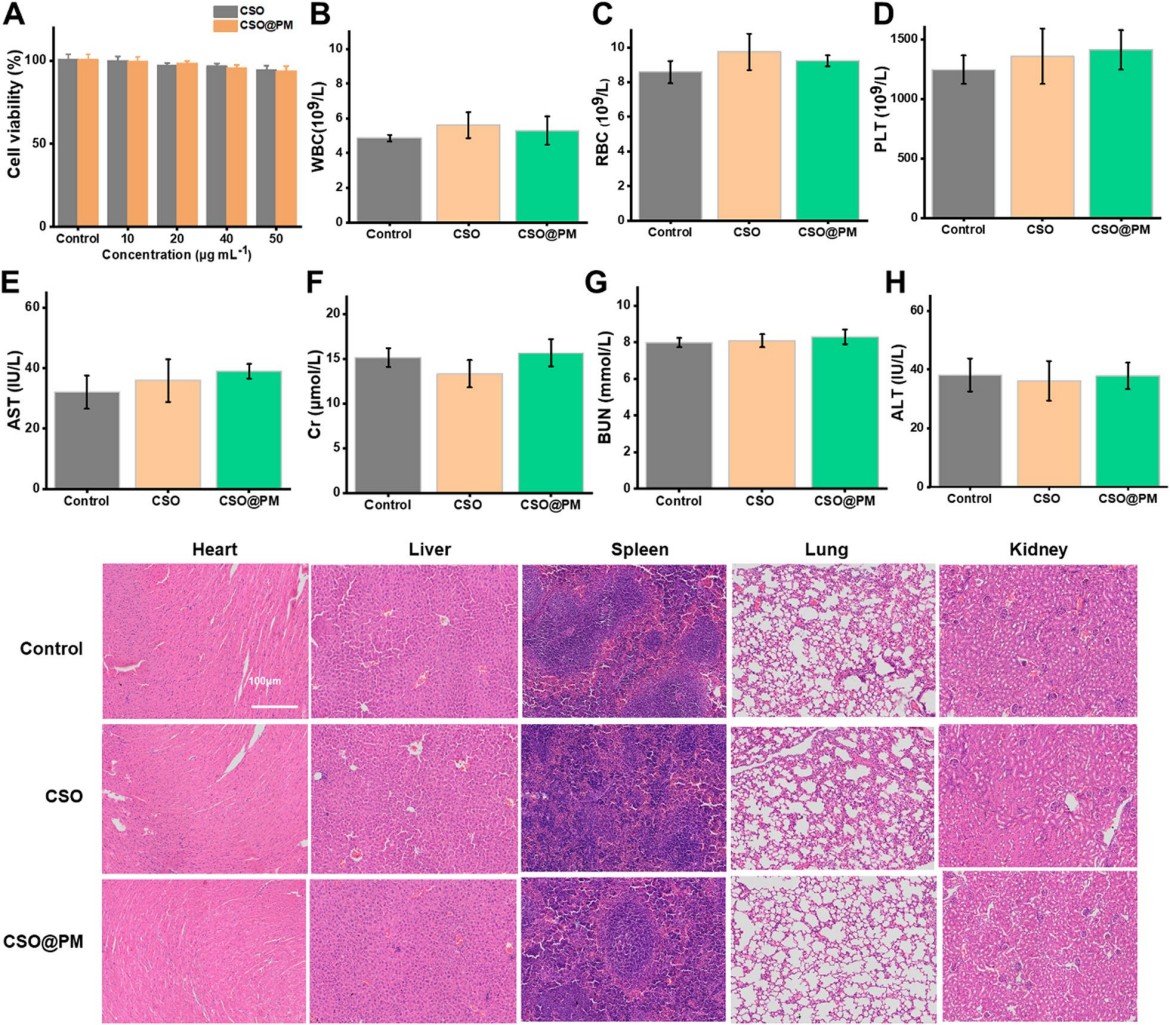
In vitro and in vivo biocompatibility of CSO@PM. **A** Cell viabilities of 3T3 fibroblasts treated with different concentrations of CSO@PM. **B**–**H** Hematology and blood biochemistry analysis results for healthy Balb/c mice sacrificed 21 days after intravenous injection with CSO@PM at a concentration of 500 mL^−1^ (*n* = 3). PBS-treated mice were used as controls. **B** White blood cells, **C** red blood cells, **D** platelets, **E** aspartate aminotransferase (AST), **F** creatinine (Cr), **G** blood urea nitrogen (BUN), and **H** alanine aminotransferase (ALT). **I** H&E staining images of the major organs (heart, liver, spleen, lung, and kidney) from mice 21 days after intravenous injection with CSO@PM (500 μg mL^−1^). Scale bar: 100 μm

Biosafety is a crucial factor for an antibacterial agent. Accordingly, the in vivo and in vitro biosafety of CSO@PM were assessed. Firstly, to assess cytotoxicity in vitro, we chose NIH-3T3 fibroblasts, which are the main components of cutaneous tissues, as the cell model [62]. NIH-3T3 fibroblasts treated with CSO and CSO@PM show similar cell viabilities (> 90%) to that for the control group after 24 h, revealing that they exhibit no obvious cytotoxicity to 3T3 fibroblasts at the test concentrations (Fig. 7A). Additionally, the hemolysis rate of a nanomaterial must be less than 5% to ensure safety during intravenous administration [63, 64]. As shown in Additional file 1: Figure S12, the hemolysis rate for 500 μg mL^−1^ CSO@PM (i.e., 10-times the concentration applied in vivo to treat infected wounds) is less than 5%. The results clearly show that the in vivo toxicity of CSO@PM at test concentrations is negligible.

Next, complete blood count (CBC) analysis was conducted to evaluate the hematological toxicity of CSO@PM in healthy mice. As shown in Fig. 7B–D, compared with the control group, there is no significant difference in blood routine index, white blood cell count, red blood cell count, and platelet count, which remain at normal levels (P > 0.05). To assess the effects of CSO@PM on visceral organs, serum enzyme level detection and histological assessment were performed. There are no significant changes in alanine transaminase (ALT), aspartate amino-transferase (AST), blood urea nitrogen (BUN), and creatinine (Cr) levels, which are indicators of liver and kidney function (Fig. 7E–I). No damage or appreciable abnormalities of the main organs (kidney, lung, spleen, liver, and heart) are observed 21 days after CSO and CSO@PM injection at 500 μg mL^−1^. This indicates that CSO@PM will have no side effects in the process of bactericidal therapy.

Overall, the excellent biocompatibility of CSO@PM makes it a very promising antibacterial agent for biomedical applications.

## Conclusions

CSO@PM, a new multifunctional antibacterial platform comprising a CSO core wrapped in a PM shell, was developed as an effective nanoagent for the treatment of bacteria-infected wounds. The PM coating significantly improves the bacteria-targeting properties of CSO, and combined with NIR irradiation efficiently kills the targeted bacteria. Furthermore, the mesoporous structure of CSO allow the CSO@PM to adsorb toxins secreted by bacteria, significantly reducing wound inflammation. Additionally, CSO@PM also stimulates re-epithelialization and granulation tissue formation, thus promoting skin tissue healing. Therefore, CSO@PM is a promising antibacterial agent for biomedical applications.

## Supplementary Information

**Additional file 1.** Additional figures and tables.

## Data Availability

All data generated or analyzed during this study are included in this published article.
