# Supplementary material for: Integrated endotoxin-adsorption and antibacterial properties of platelet-membrane-coated copper silicate hollow microspheres for wound healing
Source: J Nanobiotechnology. 2021 Nov 22;19:383. doi: 10.1186/s12951-021-01130-w (PMC8607565; doi:10.1186/s12951-021-01130-w)
Supplement: Supplementary file 1 — Additional file 1. Additional figures and tables. [file 12951_2021_1130_MOESM1_ESM.docx]

**Supporting Information**

**Integrated Endotoxin-adsorption and Antibacterial Properties of Platelet-membrane-coated Copper Silicate Hollow Microspheres for Wound Healing**

Zaihui Peng^1^, Xiaochun Zhang^2^, Long Yuan^1^, Ting Li^2^, Yajie Chen^3^, Hao Tian^1^, Dandan Ma^1^, Jun Deng^3^*, Xiaowei Qi^1^*, Xuntao Yin^2^*

1. Department of Breast Surgery, Southwest Hospital, Army Medical University, Chongqing 400038, China
2. Department of Radiology, Guangzhou Women and Children's Medical Center, Guangzhou Medical University, 510005, Guangzhou, China
3. Institute of Burn Research, Southwest Hospital, State Key Lab of Trauma, Burn and Combined Injury, Chongqing Key Laboratory for Disease Proteomics, Army Medical University, 400038 Chongqing, China.

* Corresponding authors: X.T. Y. ([xuntaoyin@gmail.com](mailto:xuntaoyin@gmail.com)); XW. Q. ([qxw9908@foxmail.com](mailto:qxw9908@foxmail.com)); J. D ([djun.123@163.com](mailto:djun.123@163.com));


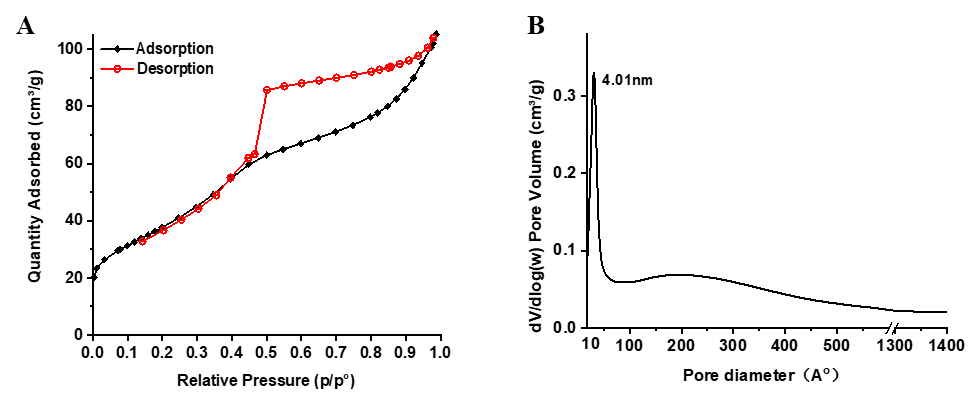


**Figure S1.** (A)Nitrogen adsorption–desorption isotherm and (B)pore size distribution patterns of CSO@PM particles.


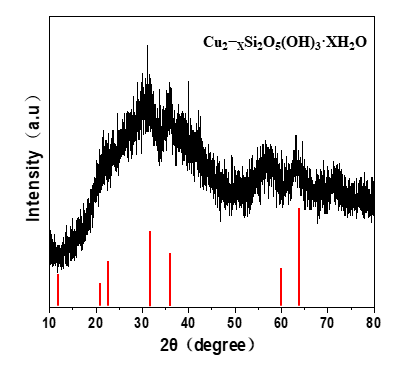


**Figure S2.** XRD pattern of CSO


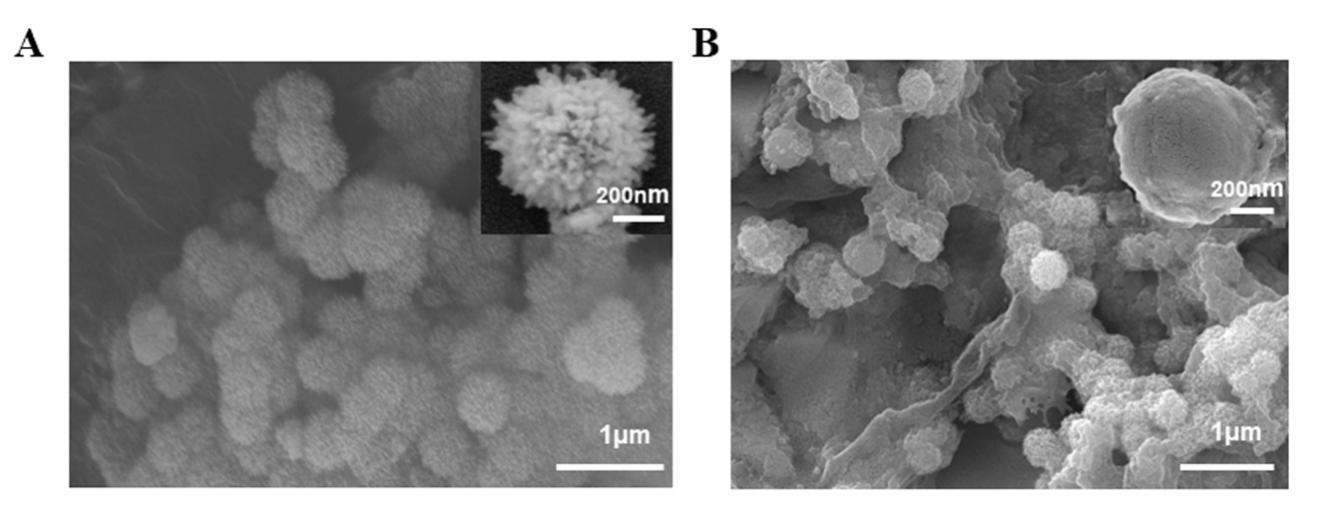


**Figure S3.** (A) SEM images showing the morphologies of CSO before and (B) after PLT membrane coating.

**Figure S4.** Change in zeta potential of bare CSO and CSO@PM in PBS for 7 days; n = 3.


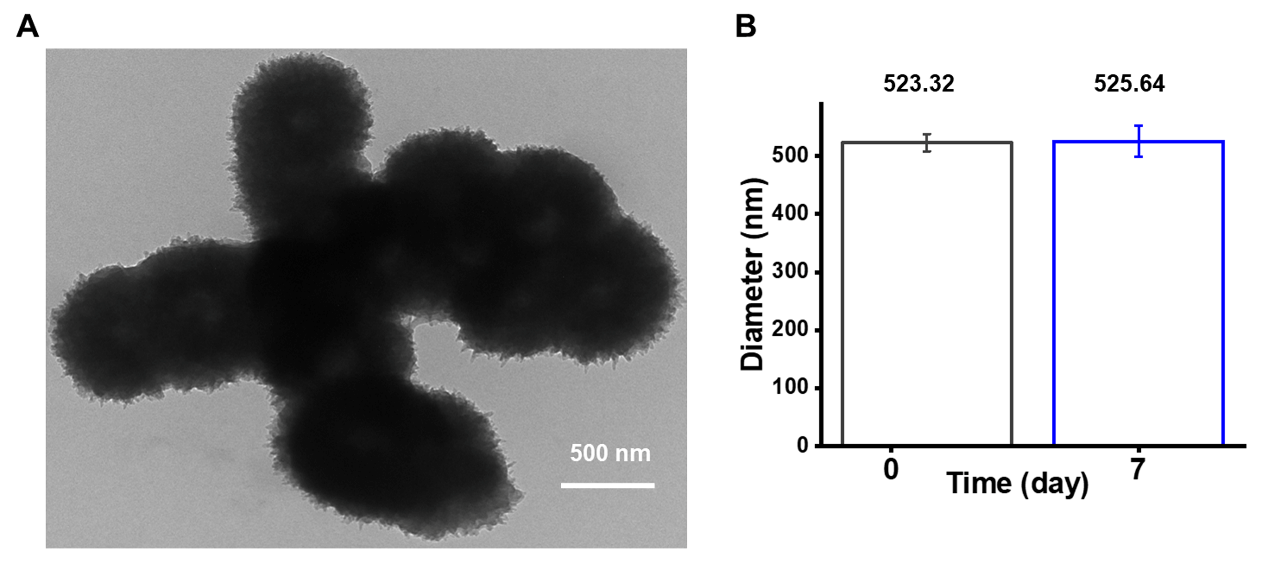


**Figure S5.** (A)TEM images of the morphologies of CSO@PM in PBS at 7 days. (B) Change in hydrodynamic diameters of CSO@PM in PBS for 7 days. Data are mean ± SD (n = 3).


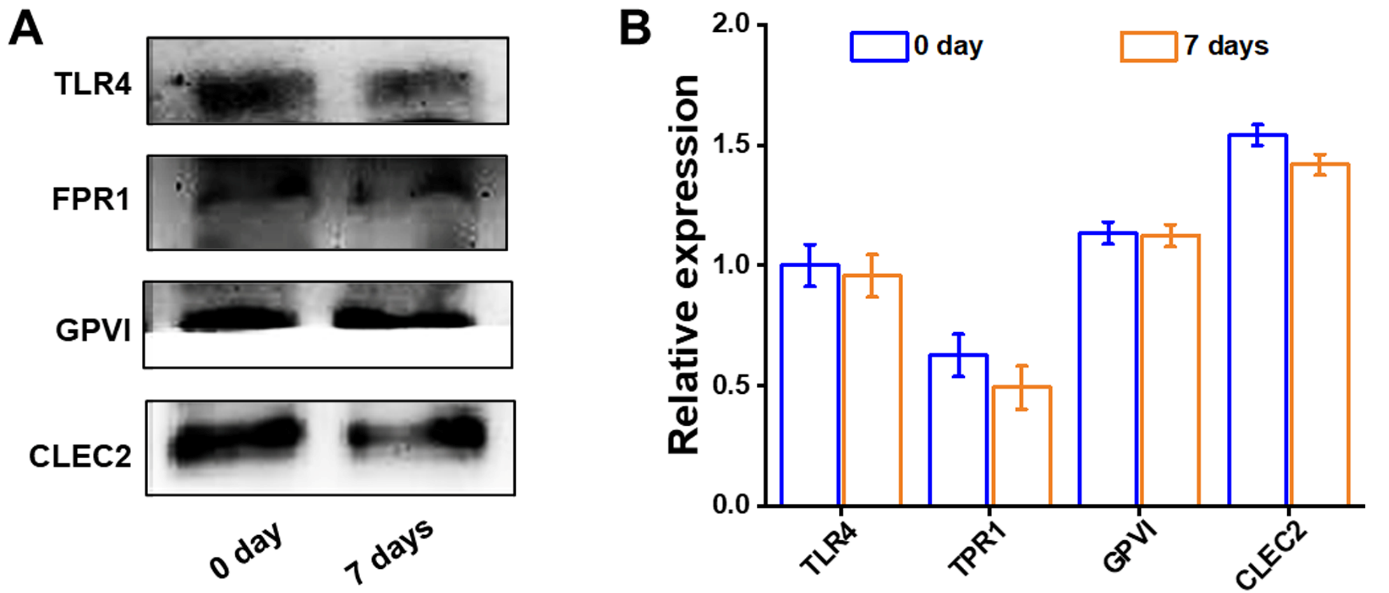


**Figure S6.** (A) Western blot analysis for TLR4, FRP1, GPVI and GLEC-2 in CSO@PM at 0 day and 7 days. (B) Relative expression of TLR4, FRP1, GPVI and GLEC-2 in CSO@PM at 0 day and 7 days. Data are mean ± SD (n = 3)


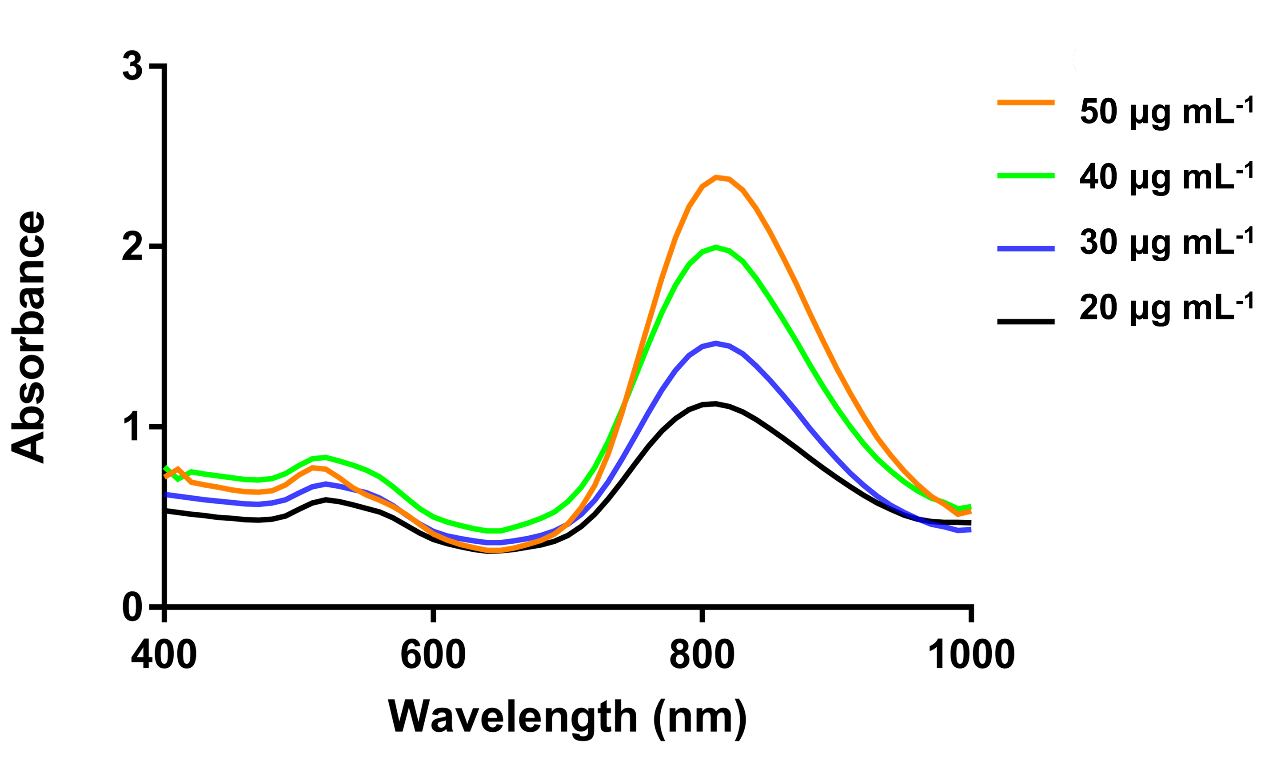


**Figure S7.** UV–vis–NIR spectra of different concentrations of CSO.


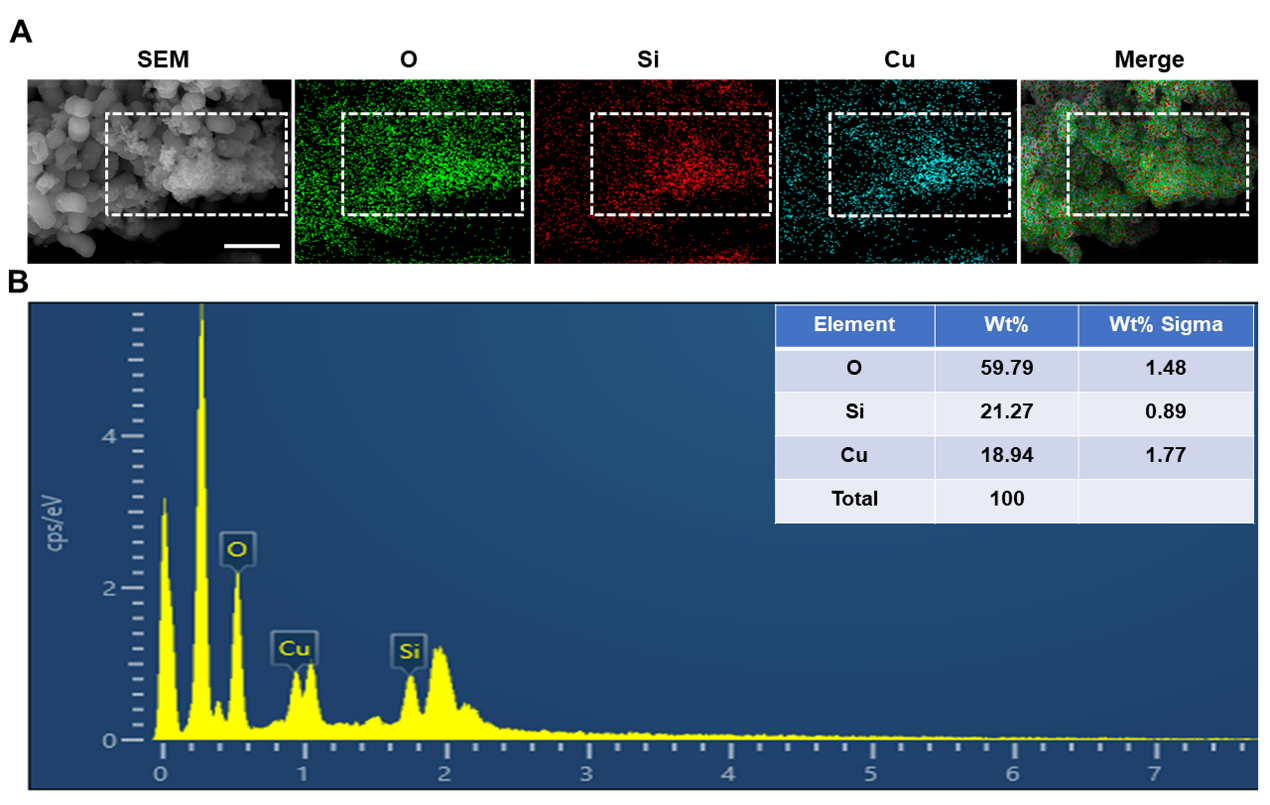


**Figure S8.** (A, B) EDS element maps and spectrum of *P. aeruginosa* treated with NPs upon laser in SEM observation indicating the element composition is O, Cu and Si

(The inserted table shows the mass percentage of elements; bar = 2.5 μm; Cps/ev: counts per second per electron-volt).


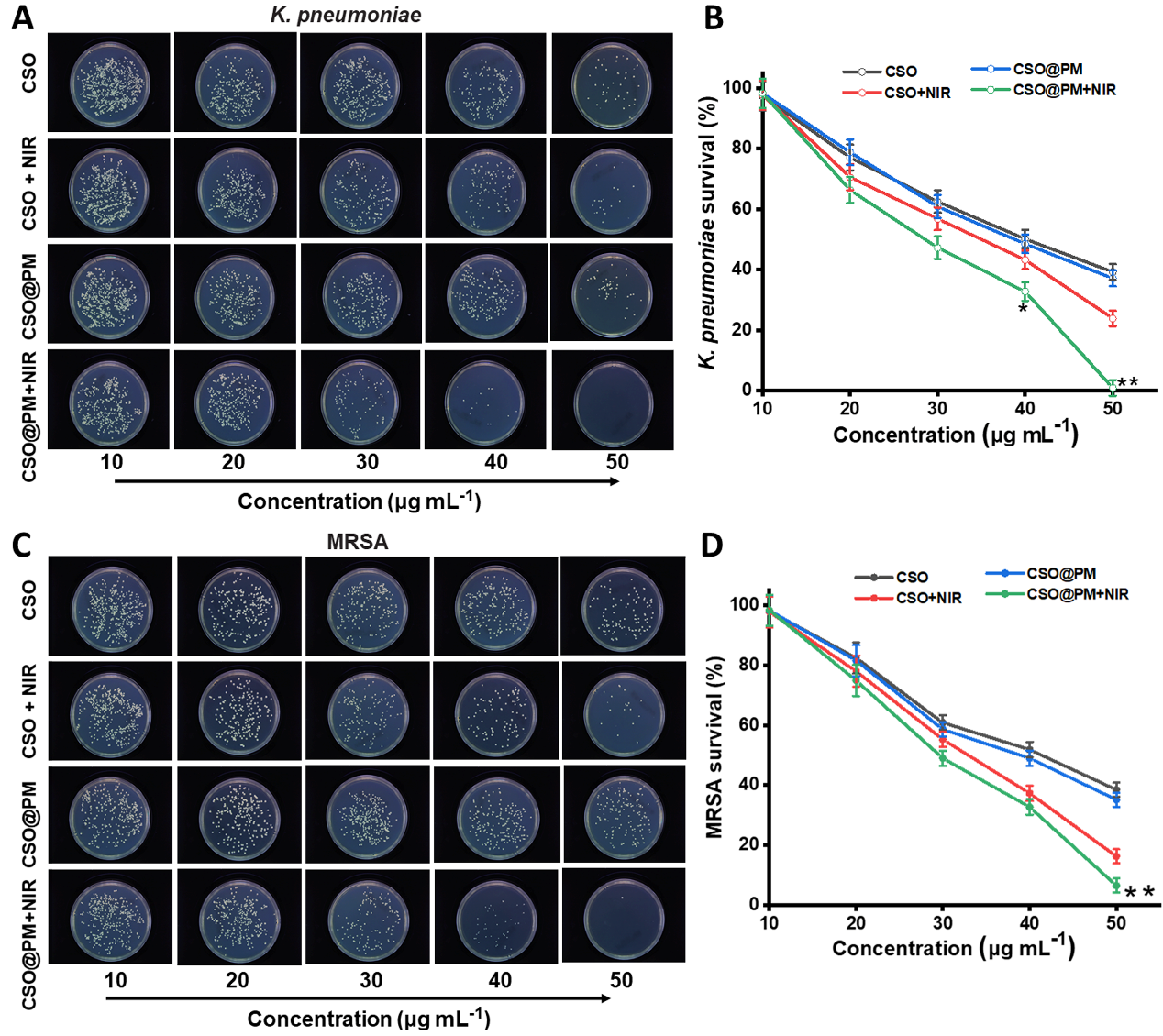


**Figure S9.** (A, C) Representative images and (B, D) quantitative analysis of bacterial colonies formed by K. pneumoniae/ MRSA after exposure to CSO and CSO@PM with or without 808 nm NIR irradiation. The values are shown as mean ± SD (n = 3) and ** indicates P < 0.001 compared with the corresponding CSO, CSO+NIR and CSO@PM group.


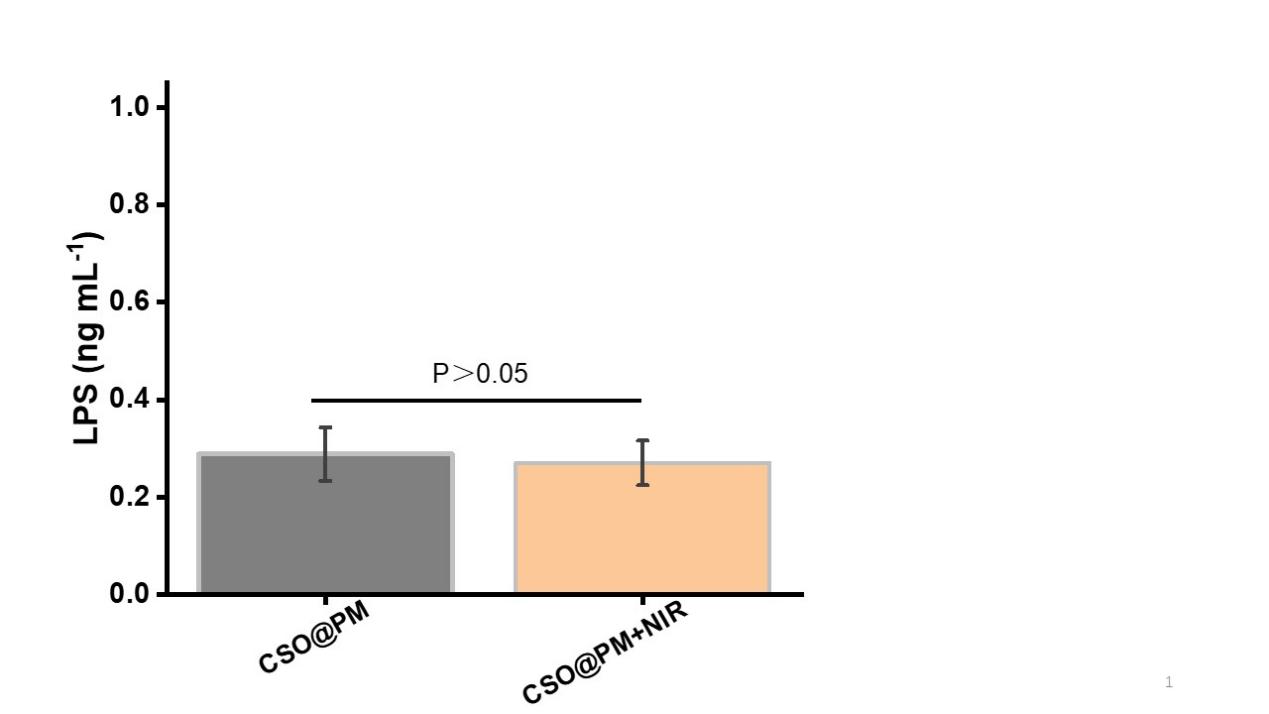


**Figure S10.** LPS adsorption for the CSO@PM (50 ng mL^−1^) and CSO@PM+NIR (50 μg mL^−1^) groups.


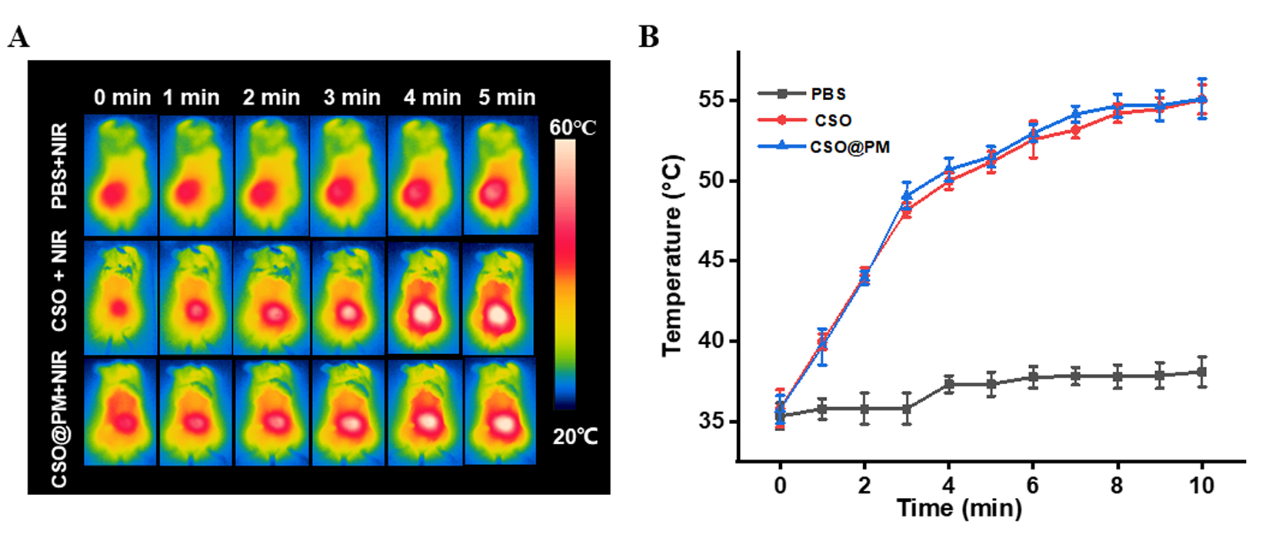


**Figure S11.** (A) Thermal infrared image of temperature evolution on dosed mice upon NIR laser irradiation (50 μg mL^-1^, 1.5 W cm^-2^). (B) Temperature evolution profile of dosed mice upon NIR laser irradiation.


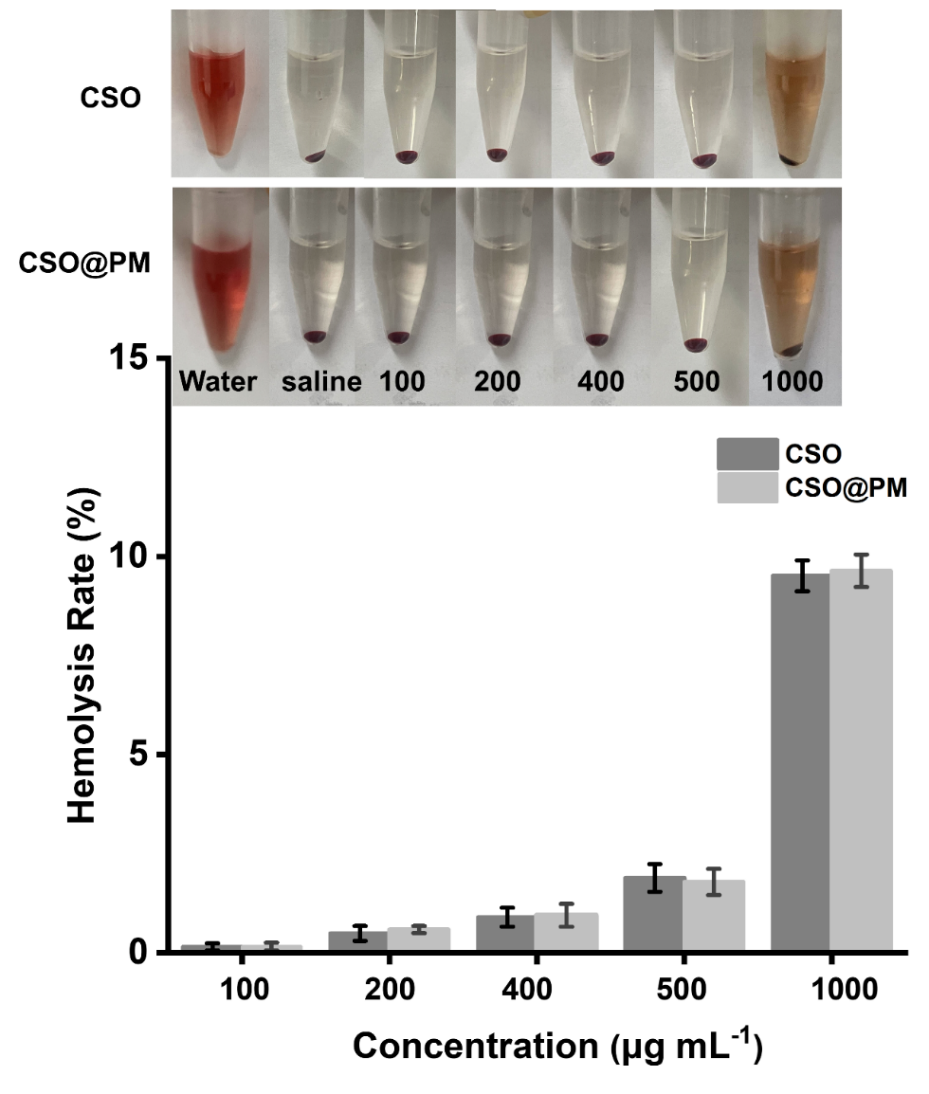


**Figure S12.** *In vitro* hemolysis test of CSO/CSO@PM. Data represent means ± SD (n=3).

**Table S1. Primer sequences for qRT-PCR.**

| **Gene** | **Forward (5’-3’)** | **Reverse (5’-3’)** |
| --- | --- | --- |
| *Il-1β* | CCTTTTGACAGGTCAGTGGGT | GATGAGTTGGGGACTCTCTGG |
| *Il-6* | TCCTTCCTACCCCAATTTCCA | GCACTAGGTTTGCCGAGTAGA |
| *Actin* | TGCTGTCCCTGTATGCCTCTG | TGATGTCACGCACGATTTCC |
